# Evaluation of Cr(VI) Removal from Tanning Effluents Using Magnetic Nanoparticles of Fe_3_O_4_ Synthesized with *Olea europaea* Bone Extract

**DOI:** 10.3390/molecules29020534

**Published:** 2024-01-22

**Authors:** Maria Bejarano-Meza, Fabricio Eduardo Deza-Carrasco, Sofia Salinas-Herrera, Susan Flores-Calla, Hugo Guillermo Jimenez-Pacheco

**Affiliations:** Universidad Católica de Santa María, Urbanization San Jose s/n, Umacollo, Arequipa 04013, Peru; 72192130@ucsm.edu.pe (F.E.D.-C.); 75363738@ucsm.edu.pe (S.S.-H.);

**Keywords:** magnetic nanoparticles, magnetite, olive extract, chromium removal, tanning effluent

## Abstract

The tanning industry generates effluents with high chromium content, which require treatment prior to discharge into the sewage system. This article explores the use of magnetic magnetite nanoparticles (MNPs) to remove Cr(VI) from aqueous solutions, such as tanning effluents. The MNPs were synthesized by coprecipitation reaction using the *Olea europaea* extract as a reducing agent. Subsequently, they were characterized by dynamic light scattering spectroscopy (DLS), scanning electron microscopy (SEM) and energy dispersive X-ray spectroscopy (EDS). MNPs with irregular morphology and diameters ranging from 73.28 to 162.90 nm were obtained. Cr(VI) removal was performed using jar test methodology, and its efficiency was evaluated in the laboratory for different initial Cr(VI) (mg/L) concentration and nanoparticle (g/L) concentration. A kinetic study was developed and indicated that the equilibrium adsorption mechanism corresponds to a pseudo-second-order model. Furthermore, the isotherm analysis revealed that chromium adsorption best fits the Langmuir isotherm. Finally, Cr(VI) removal rates from 85% to 100% were achieved in tanning and retanning effluents.

## 1. Introduction

The toxic waste generated makes the tanning industry one of the most polluting industries [1]. During leather production, different chemical inputs and water volumes between 50 and 100 L per kg of salted leather are used [2], which generates wastewater with high concentrations of pollutants, including heavy metals such as chromium, which, in its hexavalent form (VI), becomes a contaminant due to its high mobility and solubility in aqueous media, tends to bioaccumulate and has a carcinogenic effect [2,3].

In some cities, such as Arequipa in the Rio Seco Industrial Park (PIRS), tanning effluents are discharged into a sewage system or river, causing contamination of the environment and flowing into bodies of water.

These effluents have high concentrations of toxic heavy metals, especially hexavalent chromium (Cr(VI)). It also stands out that Cr(VI), due to its high toxicity associated with high mobility and solubility in aqueous media, tends to bioaccumulate, which can lead to serious effects on human health, such as carcinogenic effects [3]. Nowadays, various environmental problems can be addressed by nanotechnology. Metal and metal oxide nanoparticles, due to their more efficient adsorption and reduction capabilities than their macro or micro counterparts, have shown outstanding properties for adsorption/reduction of a large number of heavy metal ions, such as Ni^2+^, Cu^2+^, Cd^2+^, Zn^2+^ and Cr^6+^, and catalytic degradation of some organic pollutants [4,5].

The synthesis of metal and metal oxide nanoparticles has been carried out using conventional methods and green synthesis. Generally, the conventional methods use toxic materials, such as solvents and surfactants, which can accumulate in the environment and pollute it. This has motivated the research and development of new, more reliable and sustainable strategies for the synthesis of nanoparticles known as green synthesis [6]. A feature common to the methods of green synthesis is to reduce the use of toxic inputs in the production of nanoparticles by replacing them with clean precursors, such as plants, bacteria, actinomycetes, fungi, yeasts, viruses, among others [7]. Among these precursors, Moringa oleifera leaves have been used for the removal of lead from an aqueous source, Catharanthus roseus leaves for the removal of cadmium and chromium, date palm bone for the degradation of methylene blue and Ipomoea aquatica roots for the adsorption of methyl violet, among others. The bone of *Olea europaea* stands out due to its capacity to act as a biosorbent of heavy metals, as well as the presence of various polyphenols in its composition, which make it feasible to use it as a reducing agent in the production of magnetic nanoparticles. The advantages of green synthesis of metal nanoparticles not only reduce the environmental impact but also exhibit high economic efficiency compared to some conventional methods. Other advantages include reduction in energy consumption, reduction in chemical inputs and ease of implementation on a large scale [8].

Among the metal oxide nanoparticles, iron oxides are one of the most applied because they exist in various forms in nature, such as magnetite (Fe_3_O_4_), maghemite (γ-Fe_2_O_3_) and hematite (α-Fe_2_O_3_). In recent years, different authors have reported the efficiency of iron-based nanoparticles in the removal of heavy metals, such as Cr(VI), Pb(II), Cd(II) and As(III), from aqueous solutions [9]. Magnetite has been shown to be more useful in different applications [10]. Magnetite structures at the nanoscale, called nanoparticles, have attracted much interest due to their unique magnetic property known as superparamagnetism; this property is present in particles with a size of order of magnitude of nanometers, having a behavior similar to ferromagnetic and ferrimagnetic materials, [10] being the main characteristic, which allows the MNP to be very efficient in the adsorption of heavy metals, such as chromium [11]. Aftabtalab et al. [12] discussed Cr(VI) removal from wastewaters using high adsorption iron oxide (Fe_3_O_4_) nanoparticles synthesized by the sol–gel method. In a more recent article, Hao et al. [13] showed that a green tea extract was successfully used to prepare iron nanoparticles (nFe) to remove Cr(VI) from aqueous solutions.

Accordingly, this article reports the green synthesis of magnetite (Fe_3_O_4_) nanoparticles using olive pit extract. In addition, the efficiency of these nanoparticles in Cr(VI) removal is evaluated by varying the initial Cr(VI) (mg/L) concentration and nanoparticle (g/L) concentration. The material characteristics of the nanoparticles are also presented, as well as the adsorption behavior, kinetic and isotherm models.

In general, the present research provides a sustainable alternative to contribute to solving the problem of the lack of treatment of Cr(VI) loaded effluents from tanneries before discharge into the environment. The results obtained show that the development and application of nanoparticles obtained by green synthesis using *Olea europaea* bone extract as a reducing agent are an efficient treatment for the adsorption of Cr(VI) from tanning effluents.

## 2. Results and Discussion

### 2.1. Characteristics of the Tanning and Retanning Effluents

The tanning and retanning effluent samples were analyzed in the laboratory. Table 1 shows the physico-chemical characteristics of the samples.

The results indicate that for the retanning effluent, the parameters of pH, total chromium, hexavalent chromium and total suspended solids exceed the maximum permissible limits (MPL). In the case of the tanning effluent, the parameters of total chromium, hexavalent chromium and chemical oxygen demand (COD) exceed the maximum permissible limits (MPL), with total chromium and hexavalent chromium being the most relevant parameters in this study. These parameters exceed the MPL by almost 42% in the case of total chromium, 1% for hexavalent chromium in the retanning stage and 258% in the tanning stage.

### 2.2. Favorable Operating Conditions and Validation of the Method for Cr(VI) Quantification

#### 2.2.1. Linearity

**Figure 1:**
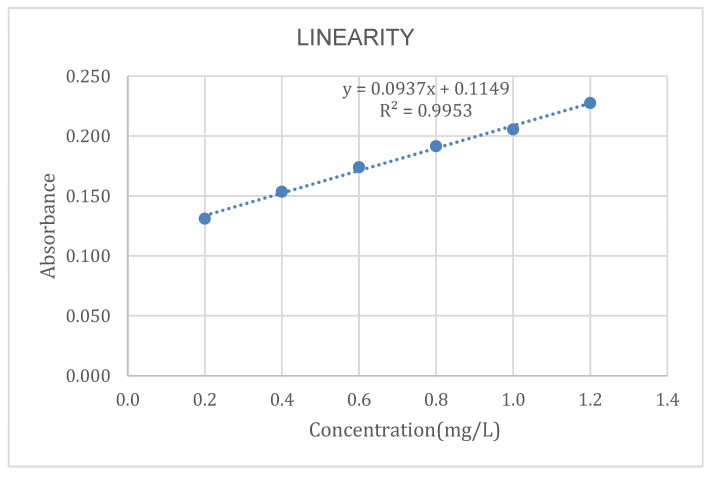
Calibration curve.

To determine the linearity of the method, a calibration plot of concentration vs. absorbance is presented in Figure 1.

The R^2^ value obtained from the equation of the line is 0.9953, which is very close to the value obtained by Pacheco-Portugal [14], whose R^2^ was 0.9964. Similarly, an R^2^ value of 0.9996 was reported by Cañazaca and Ccama [15] in the validation of Cr(VI) in wastewater.

According to international standards (AOAC), values greater than 0.99 are considered acceptable; therefore, we conclude that our method is linear.

#### 2.2.2. Precision and Repeatability

To determine the precision and repeatability of the method, the measurement of the analyte Cr(VI) was performed in triplicate under the same working conditions and with the same instrument. The results are shown in Table 2.

According to the ICH validation method, the acceptance criterion for repeatability and precision is that the %RSD is less than 2%; therefore, comparing this criterion with our results, it is concluded that the method is precise and accurate. The RSD value obtained is 0.023%, which is below 2%.

#### 2.2.3. Detection Limit (LOD) and Limit of Quantification (LOQ)

The detection limit allows finding the lowest concentration, which can be detected with statistical significance using the analytical procedure studied.

The results obtained with the calculated values for both the limit of detection and the limit of quantification are shown in Table 3.

Based on the results obtained, shown in the table above, it is established that the method can be used for samples whose Cr(VI) concentration is above 0.0528 mg/L and quantifies samples whose concentration is equal to or greater than 0.1760 mg/L.

#### 2.2.4. Accuracy

The accuracy was determined by calculating the percentage of recovery (% R); the results obtained are shown in Table 4.

According to the ICH validation method, the acceptance criterion for accuracy is 95% < Percent Recovery < 103%; therefore, it is considered that the validated analytical method is accurate, since the recovery percentage of 102.949 is within the range of the acceptance criterion.

### 2.3. Characteristics of the Magnetite Nanoparticles (MNPs)

Dynamic light scattering (DLS) is a hydrodynamic technique, which provides insight into the particle size distribution in a liquid system. The measurement is based on the detection of variations in the intensity of light scattered by particle scattering. The DLS results revealed that most of the MNPs had a size of 161.8 nm, corresponding to 98.5% of the total population, while a smaller population, corresponding to 1.5%, presented a size of 5362 nm.

**Figure 2:**
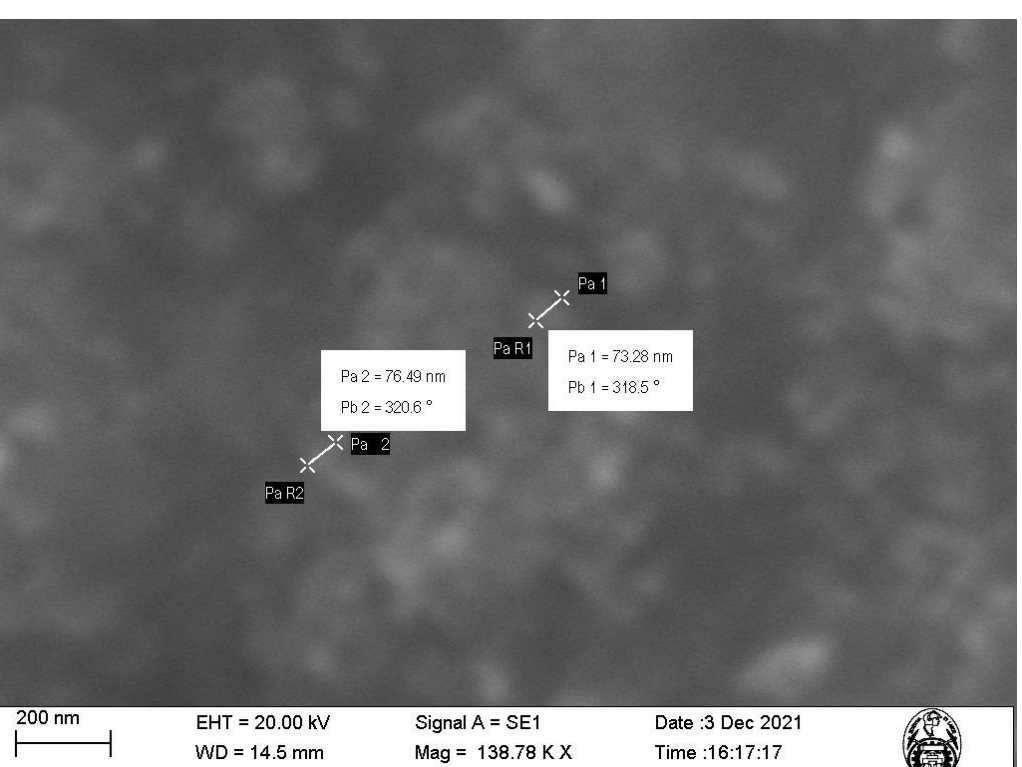
SEM image of the magnetite nanoparticles (MNPs) at 138.78 K magnification.

**Figure 3:**
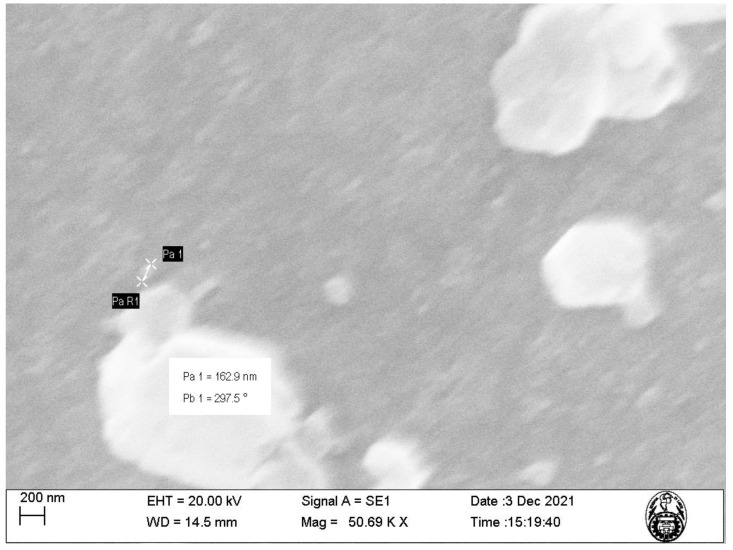
SEM image of the magnetite nanoparticles (MNPs) at 50.69 K magnification.

The characterization of nanoparticles using SEM (Figure 2 and Figure 3) showed that the nanoparticles exhibit sizes within the range of 73.28–162.9 nm and an irregular shape presenting agglomerations (Figure 3). This is due to the synthesis method of the nanoparticles using plant extract, which is a mixture of reducing and stabilizing agents with complex chemistry, producing heterogeneous MNPs and not homogeneous, as is the case when using chemical agents [16]. Such heterogeneity is an advantage, given that it facilitates the adsorption process by providing active sites for physical exchanges and chemical reactions, as discussed by Es’haghi et al. [17], who synthesized indefinitely shaped and highly agglomerated magnetic iron nanoparticles coated with olive oil.

Our results show that, although the particle diameter found with DLS is within the range measured by SEM, the difference in the measurements can be attributed to the formation of a hydrodynamic radius characteristic of dilute solutions, such as those used for DLS characterization, while for SEM, the sample used is completely dry, which allows for a much more accurate measurement.

**Figure 4:**
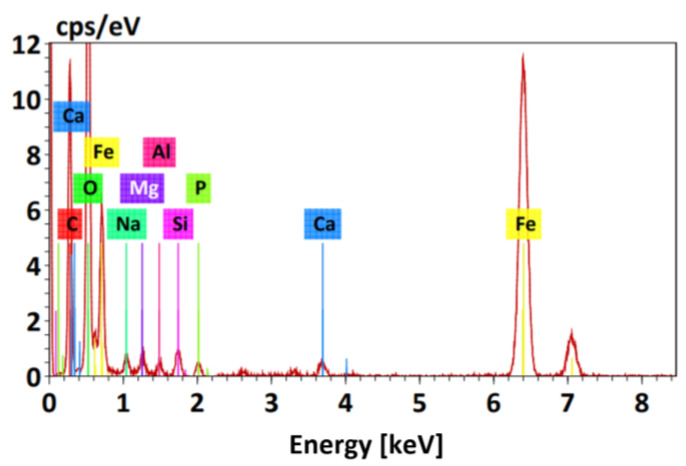
EDS spectrum of magnetite nanoparticles (MNPs).

Figure 4 shows the EDS spectrogram of the nanoparticles. The presence of elemental iron with an atomic percentage of 9.4% can be observed, corresponding to the intense peak between 6 keV and 7 keV. The presence of oxygen is also detected with an atomic percentage of 43.72%, corresponding to the most intense peak between 0 keV and 1 keV, suggesting the oxidation of nanoparticles due to their exposure to water or air. Peaks of trace element contamination are also observed, attributed to the sample holder.

### 2.4. Removal Efficiency of Cr(VI)

After the experiments, it was observed that the optimal operating pH was pH = 2, which achieved a removal percentage of 100%, while with a basic pH, only 30% removal was achieved. These findings corroborate those obtained by Fazlzadeh et al. [18] and Assi et al. [19] and can be attributed to the fact that pH determines the adsorption of metal ions, since it modifies the surface charge of the sorbent; therefore, it modifies the stability of hexavalent chromium, causing its reduction and turning it to trivalent chromium.

The equilibrium time at which the adsorption process became constant was 30 min, where the removal percentage was 100%, remaining constant and registering a stagnation in the adsorption process.

The optimum agitation speed was 140 rpm, and a removal of 90% was obtained. This was the maximum value reached with the jar equipment used, indicating that the higher the agitation speed, the higher the percentage of Cr(VI) removal.

With the adsorption process using jar tests, removal percentages of 100% for the initial concentration of Cr(VI) of 10 mg/L and 20 mg/L were reached. On the other hand, for 50 mg/L, percentages of only up to 75% were reached due to the saturation of the MNPs, which reached their highest performance with concentrations of up to 20 mg/L. The most efficient combinations were obtained with initial concentrations of Cr(VI) of 10 mg/L and 20 mg/L and a nanoparticle concentration of 1 g/L. These values are in accordance with the values obtained by Hao, Li, Zhang and Jiao [13], who achieved a removal efficiency of 91.6%.

The effect of a factor can be defined as the observed change in the response variable (% removal) due to the change in the level of that factor. It should be noted that the main effects are the changes in the mean value of the response variable, which is due to the individual action of each factor. Based on the Pareto diagram obtained using the OriginLab program, it can be evidenced that the initial concentration of Cr(VI) is a limiting factor for the nanoparticles, since these will saturate faster, thus limiting their adsorption capacity when the initial concentration of the pollutant increases. However, the statistical analysis also allowed us to determine that the performance of MNP in contaminant removal has a relationship directly proportional to MNP concentration.

In relation to the percentage of Cr(VI) removal from tanning and retanning effluents, filtration was initially carried out before the removal process using magnetite nanoparticles to eliminate suspended solids in the tanning effluent sample. The results obtained from the process showed a removal of 85% and 100% for the tanning and retanning effluents, respectively, since the tanning sample showed a higher initial Cr(VI) concentration. Following the adsorption process, the nanoparticles were recovered with a magnet to be characterized by EDS and to determine their final composition, obtaining mainly iron with a presence of 76.35%.

### 2.5. Isothermal Models

#### 2.5.1. Langmuir Isotherm

**Figure 5:**
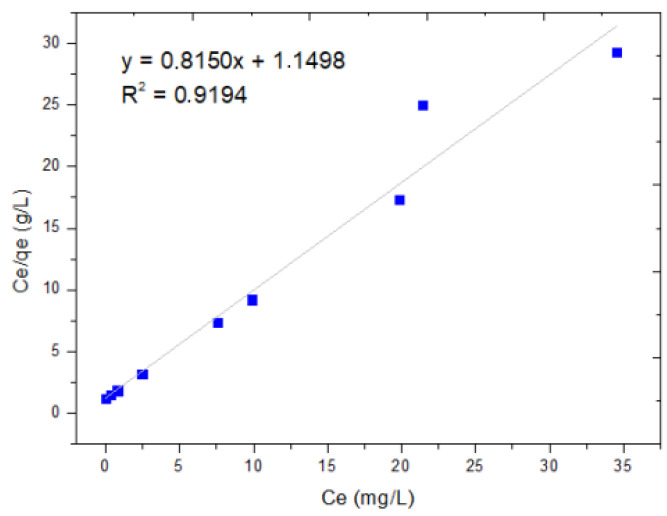
Langmuir isotherm.

The Langmuir and Freundlich adsorption isotherm models were used to evaluate the experimental data of the adsorption equilibrium of dichromate ion adsorption using MNPs. Figure 4 shows the graph of the experimental values of the specific adsorption (C_e_q/q_e_) as a function of the concentration of the metal ion in equilibrium (C_e_q) and the linear form of the Langmuir equation. These results show an $R^{2}$ of 0.9194, representing the dispersion around the regression line, indicating that the phenomenon has monolayer adsorption, as shown in Figure 5.

The linearization of the isotherm using the Langmuir equation comes from the clearance of the equation; it is based on relating the values of C_e_ and C_e_/q_e_ in a linear regression of these points in order to obtain the slope and the intercept, which will allow us to find the values of q_max_ and b.

(1) $$\frac{C_{e}}{q_{e}}=\frac{1}{bq_{max}}+\frac{1}{q_{max}}C_{e},$$

where C_e_: adsorbate Cr(VI) equilibrium concentration in solution (mg/L); b: constant referring to the adsorption affinity between the adsorbent (nanoparticle) and adsorbate Cr(VI); q_max_: the maximum amount of sorbate Cr(VI) per unit mass of adsorbent (nanoparticulate) to complete the monolayer (mg/g).

In Table 5, the value of the equilibrium parameter (R_L_) indicates the type of isotherm.

To calculate the value of q max, which is the inverse of the slope, the following result is obtained: 1.226 y b=0.709. To calculate R_L_, each C_0_ concentration value of 0, 10, 20, 50 and 80 is replaced:

(2) $$R_{L}=\frac{1}{1+bC_{0}},$$

According to Table 6, the R_L_ values are less than zero, except for C_0_ of 0 mg/L, with an average R_L_ of 0.2468, which indicates that the absorption is favorable compared to the R_L_ values in Table 5, where the types of isotherms are found.

#### 2.5.2. Freundlich Isotherm

**Figure 6:**
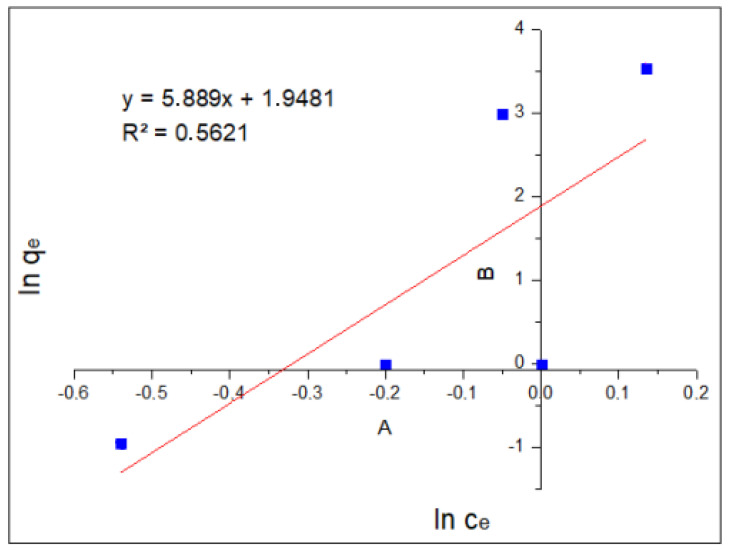
Freundlich isotherm.

Figure 6 shows the plot of log(q_e_) as a function of log(C_e_) and the linear form of the Freundlich equation. These results show an $R^{2}$ of 0.5621, which represents or gives indications of multi-layer adsorption.

(3) $$q_{e}=K_{f}*C_{e}^{1/n},$$

where C_e_: the Cr(VI) concentration at equilibrium in (mg/L); K_f_: the Freundlich counter, which is related to the incorporation of all factors affecting adsorption capacity (it is a parameter known to be related to bond strength and adsorption, as well as adsorbent capacity); n: an indication of favorability of metal adsorption by the nanoparticles.

Linearization using Freundlich’s method focuses on relating the values of log(C_e_) and log(q_e_) in a linear regression of these points in order to obtain the slope and the intercept, which will allow us to obtain the values of K_f_ and n.

(4) $$\log\left(q_{e}\right)=\log\left(K_{f}\right)+\frac{1}{n}*log(C_{e}),$$

The values of K_f_ and 1⁄n are obtained from the intercept and the slope, resulting from plotting log(q_e_) vs. log(C_e_). The constant “n” is an indication of metal adsorption favorability by the nanoparticles.

The value 1/n is the slope related to surface heterogeneity and bond distribution. It is dimensionless and is related to the intensity of adsorption. In general, in systems following the Freundlich isotherm, adsorption occurs with the formation of multi-layers instead of only a monolayer, which is the case in the Langmuir isotherm.

To calculate the values of 1/n and K_f_, the Freundlich equation is cleared with the values of the linear regression found. To calculate the value of K_f_, the antilogarithm of the intercept value is used.

$$Log{(K}_{f})=0.9018\;K_{f}=7.967\;n=0.1698$$

The Langmuir isotherm model was obtained by graphing C_e_/q_e_ vs. C_e_, resulting in an equation Y = 0.815X + 1.1498 with a correlation coefficient R^2^ = 0.9194, while the Freundlich model was obtained by graphing log(q_e_) vs. log(C_e_), as shown in Figure 5, obtaining an equation Y = 5.889X + 1.9481 with a correlation coefficient R^2^ = 0.5621. When comparing the correlation coefficients (R^2^ = 0.0.9194 vs. R^2^ = 0.5621), it is observed that the Langmuir model shows a better fit with the data, since it has the highest value. This means that our process is limited to monolayer adsorption, and there is no interaction between the adsorbed particles.

The q_max_ value was 1.226 mg/g. This determined the number of moles of solute required to form a monolayer on the surface of the sorbent. While the value of b was 0.709—which indicates that the adsorption of Chromium (VI) is favorable, since increasing the value of variable b causes the adsorption strength to increase proportionally—this result was similar to that obtained by Cañazaca and Ccama [15], who obtained in their adsorption process of Cr(VI), using zerovalent nanoparticles, a fit of their data with the Langmuir isotherm with a value of b of 1.061.

Table 7 shows the parameters of the Langmuir and Freundlich models for the Cr(VI) adsorption equilibrium using the MNPs.

The results obtained show that the Langmuir model is the one, which best fits our data, presenting a coefficient of determination R^2^ of 0.9194, higher than that obtained for the Freundlich isotherm, whose R^2^ is 0.5621. The value of q_max_ was 1.226 mg/g, determining the number of moles of solute required to form a monolayer on the surface of the absorbent. We also observed that the value of b was 0.709, which indicates that the adsorption of chromium (VI) is favorable, since an increase in the value of variable b allows for the adsorption strength to increase proportionally. To evaluate adsorption according to the shape of the isotherm, the equilibrium parameter or Langmuir parameter (R_L_) is used, which must be calculated in the amplitude of the initial concentrations (C_0_) of the process.

(5) $$R_{L}=\frac{1}{1+bC_{0}},$$

C_0_ is considered as the reference concentration for equilibrium, such that C_e_ < C_0_ in the range of C_e_ concentrations. In addition, C_e_ and C_0_ must satisfy the Langmuir equation. The values taken by R_L_ are less than zero, except for C_0_ of 0 mg/L, with an average R_L_ value = 0.2468, which indicates that the adsorption is favorable compared to the values of R_L_.

### 2.6. Kinetics

The application of the kinetic models to the experimental data of the adsorption of the metal ion as a function of time allowed us to evaluate which of the proposed models best describes the kinetics and the limiting step in the adsorption of the metal ion on the adsorbent. These are shown in Table 8 and Table 9.

**Figure 7:**
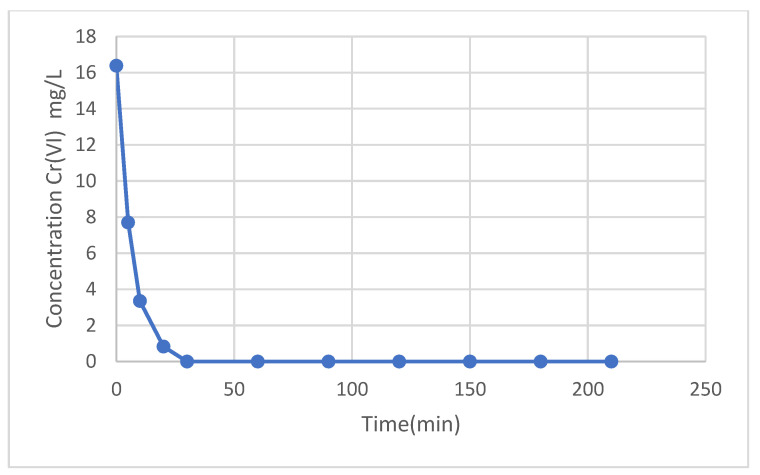
Cr(VI) concentration (mg/L) vs. contact time.

Figure 7 shows the results of the change in Cr(VI) concentration as the contact time with the magnetic nanoparticles increases. It can be seen that the equilibrium time was reached after 30 min, where the chromium concentration was 0 mg/L and remained constant from that moment on.

**Figure 8:**
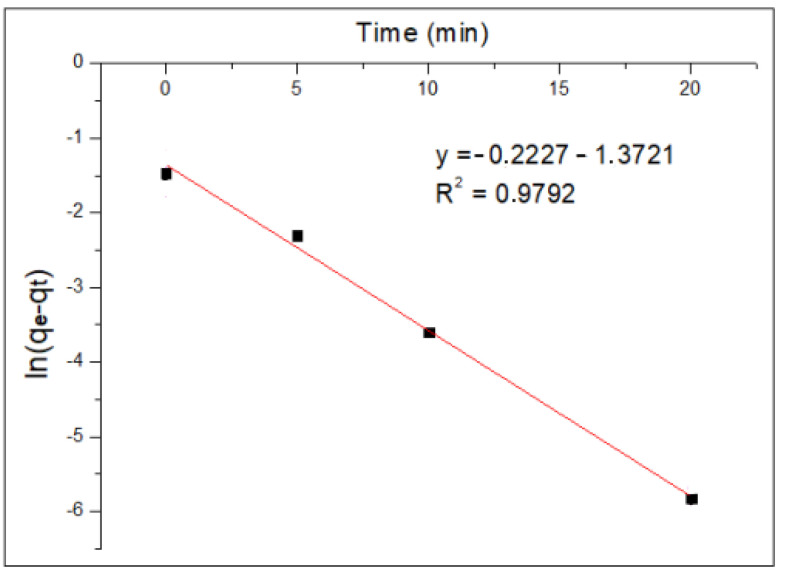
Pseudo-first-order kinetic model.

**Figure 9:**
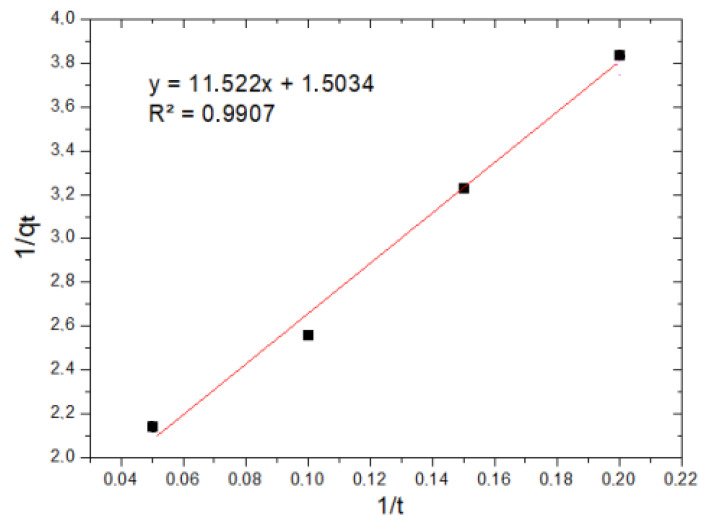
Pseudo-second-order kinetic model.

Figure 8 and Figure 9 show the application of the pseudo-first-order models and the application of the pseudo-second-order model to the experimental data of Cr(VI) adsorption kinetics using MNP, respectively.

The results show that, for the pseudo-first-order model, the R^2^ value is 0.9792, while for the pseudo-second-order model, the value of the coefficient of determination R^2^ is 0.9907. This indicates that the experimental data obtained fit the pseudo-second-order kinetic model better; therefore, it is preferable to have a chemisorption process, where the rate limiter of the adsorption process is the same reaction and adsorption and not a mass transfer of the metal ion from within the solution to the surface of the nanoparticle.

Table 10 shows the parameters of the pseudo-first-order and pseudo-second-order models. As can be noted, the adsorption velocity for the pseudo-second-order mechanism (K_2_) is higher than that obtained in the pseudo-first-order model (K_1_).

## 3. Experimental Procedure

The collection and characterization of the tanning and retanning effluents prior to their discharge into the sewage system were carried out in situ and ex situ (see Section 2.1).

### 3.1. Effluent Characterization

The collection of effluent samples from the tanning stage was carried out at the Isidro Viza tannery located in the Río Seco Industrial Park, following the *Manual for Inspectors: Industrial Effluent Control* [20]. Samples were collected before effluent discharge to the sewer and placed in previously sterilized PVC bottles for transport in a thermal container (cooler) (away from sunlight) at a temperature of 4 °C using ice (or ice pack).

In situ measurements of pH, dissolved oxygen, conductivity and temperature were performed using a portable multi-parameter WTW Model 3620.

Quantification of ex situ parameters was performed using the international standard methodology for waters and effluents APHA-AWWA-WEF 2017. The parameters of total suspended solids, hexavalent chromium, total chromium, oils and fats, chemical oxygen demand (COD) and biological oxygen demand (BOD) were measured using the methods included in the *Standard Methods for the Examination of Water and Wastewater.23rd Ed. 2017* [21].

### 3.2. Preparation of the Olive Pit Extract

For the preparation and determination of the total phenolic content of *Olea europaea* bone extract, 500 g of olive pits was washed and dried and then crushed. Then, 10 g of the crushed biological material was added to a 1:1 solution of alcohol and water and left to stand for 24 h at room temperature. The resulting solution was filtered using slow filter paper, and the alcohol was eliminated by phase separation using a rotary evaporator. The final extract obtained was used for the determination of phenolic concentration using standard methods of the American Public Health Association.

### 3.3. Synthesis of Magnetite (Fe_3_O_4_) Nanoparticles

In a beaker, 0.07 mL of 40% ferric chloride was mixed with 50 mL of ultrapure water and 0.07 g of ferrous sulfate hepta-hydrate. The mixture was stirred at 200 rpm using a shaker (model MS-H280-PRO) at room temperature for 20 min. Subsequently, this solution was brought to pH = 12 by slowly adding 2M sodium hydroxide (NaOH); then, the solution was brought to a temperature of 80 °C, raising the temperature of the shaker. The temperature was controlled with the help of a thermometer. Forty milliliters of phenolic extract was added to the solution and left in constant agitation at 200 rpm for 2 h. Then, the solution was subjected to an ultrasound field at a temperature of 70 °C for 20 min. Finally, the nanoparticles were washed 2 times in a centrifuge at a revolution of 40 rpm for 10 min using ethyl alcohol and dried at 70 °C for 8 h in an oven. The dried nanoparticles were crushed with a mortar and pestle and stored for batch characterization and adsorption experiments.

### 3.4. Characterization of the Produced Nanoparticles

The synthesized nanoparticles were characterized using different techniques. The size of the nanoparticles in solution was evaluated by dynamic light scattering spectroscopy (DLS), using the DLS-Zetasizer Malvern ZS 90. To carry out the analysis, the dry nanoparticles were ground using a mortar until obtaining a very fine powder; of this powder, 0.002 g was taken in a test tube with 10 mL of deionized water, and then, it was dispersed by sonicating the mixture for 3 min. To record the measurement, a drop of the dispersion was taken and placed in a cuvette, adding 1.5 mL of deionized water; the ready cuvette was placed in the sample holder of the equipment, and a reading was taken.

The morphology and size distribution of the nanoparticles were determined by scanning electron microscopy (SEM), using the ZEISS EVO LS10 scanning electron microscope. For the analysis, the nanoparticles were dried and crushed into a fine powder for reading. The nanoparticles were placed on the sample holder using carbon tape. The elemental composition of the nanoparticles was determined by energy dispersive spectroscopy (EDS), using Hitachi equipment (model SU-8230).

### 3.5. Favorable Operating Conditions and Validation of the Method for Cr(VI) Quantification

For the quantification of hexavalent chromium, the colorimetric method was used, using a UV-VIS spectrophotometer.

#### 3.5.1. Validation of Diphenyl Carbazide

The following solutions were prepared for validation. Chromium stock solution: Potassium dichromate (K_2_Cr_2_O_7_) was dried for 1 h in a desiccator to constant weight. Then, 0.071 g of potassium dichromate was weighed, placed in a vial and filled up to the mark with 250 mL of distilled water. This solution was brought to pH = 2 by adding 37.5 mL of 0.5% sulfuric acid for preservation. One milliliter of the solution contained one-tenth milligram of Cr(VI). 1.5-Diphenylcarbazide solution 0.5% (*m/v*): 125 mg of 1.5-Diphenylcarbazide was weighed and then dissolved in 25 mL of acetone. Sulfuric acid solution 1:1 or 50% for the method: 25 mL of concentrated sulfuric acid was taken and filled up to the mark in a 50 mL flask with distilled water. N sulfuric acid solution for pH adjustment: 0.275 mL was taken—taking into account the concentration and purity of the acid—to finally attain a final volume of 200 mL.

Subsequently, the 100 ppm Cr(VI) standard solution was prepared using 1.5-Diphenylcarbazide (known concentration of Cr(VI)) by placing 100 μL of H_2_SO_4_ 50% and 200 μL of diphenyl carbazide in a 10 mL flask, which was filled up to the mark with distilled water. Finally, spectrophotometric scanning was performed at 540 nm of the Cr(VI) solution at a concentration of 10 mg/L.

#### 3.5.2. Linearity

An absorbance vs. concentration calibration curve was constructed using 7 chromium concentrations: 0.0, 0.2, 0.4, 0.6, 0.8, 1.0 and 1.2 mg/L. Each point was evaluated in triplicate; the data were statistically processed with the linear regression test. Linear correlation coefficient (R), the coefficient of determination (R^2^), intercept (a) and slope (b) were determined with 95% confidence.

#### 3.5.3. Accuracy and Repeatability

Six measurements of 0.6 mg/L chromium concentration were recorded under the same conditions (operator, apparatus, laboratory and short time interval). In a 25 mL vial, 174 mL of chromium stock solution was used. Afterward, 2500 μL of the previous solution, 250 μL of 0.1 N sulfuric acid and 500 μL of the 1.5-Diphenyl carbazide solution were mixed in a 25 mL vial. See Table 11.

#### 3.5.4. Limit of Detection (LOD) and Limit of Quantification (LQO)

Eight blank readings (containing all reagents, except the chromium (VI) standard) were performed on the same day. The average and standard deviation of the results were then calculated. In addition, the slope found in the calibration curve used for the linearity of the method was used.

(6) $$LOD=\frac{Ybl+3DSbl}{m}\times \frac{1}{\sqrt{n}},$$

(7) $$LOQ=\frac{Ybl+10DSbl}{m}\times \frac{1}{\sqrt{n}},$$

where DSbl: standard deviation of targets; Ybl: average of blank readings; m: slope of the calibration curve; n: data number.

#### 3.5.5. Accuracy

The percentage recovery of the average absolute analyte was analyzed; three measurements were performed in triplicate at a concentration of 0.6 mg/L of chromium (VI).

### 3.6. Adsorption Experiments

#### 3.6.1. Synthetic Solutions

The adsorption efficiency of the produced magnetite nanoparticles was evaluated in jar tests using synthetic solutions and tanning and retanning effluents. The operating conditions, such as pH, equilibrium time and agitation speed, were optimized. For the adsorption process using synthetic solutions, different initial Cr(VI) concentrations (10, 20 and 50 mg/L) and different nanoparticle concentrations (0.5–1 and 2 g/L) were used. In 50 mL beakers, volumes of 30 mL of the synthetic solutions were placed, and then, different concentrations of nanoparticles were added. The samples were taken to the jar equipment under the optimized operating conditions: pH = 2, equilibrium time = 30 min, speed = 140 rpm. After the adsorption process, 0.1 mL aliquots were taken and filtered to remove the nanoparticles from the solution. Finally, 4 aliquots were read using the diphenyl carbazide colorimetric method to determine the final Cr(VI) concentration.

#### 3.6.2. Tanning and Retanning Effluents

Initially, for the adsorption process with tanning and retanning effluents, the samples were filtered under vacuum in order to eliminate particles or other suspended materials. Subsequently, three aliquots of the filtered samples of 30 mL each were taken and placed in beakers to carry out the adsorption process in triplicate using optimized nanoparticle concentrations and operating conditions. Aliquots of 0.1 mL were taken from the final solutions and filtered using a nanopore (1–100 nm) to finally quantify the Cr(VI) concentration by spectrophotometry using the diphenyl carbazide method. Based on the final Cr(VI) concentrations found in the tanning and retanning effluents, the Cr(VI) removal percentage was calculated for each of the treated effluents.

#### 3.6.3. Thermodynamic Study

In order to obtain the isotherms, synthetic chromium solutions (0, 10, 20, 20, 50 and 80 mg/L) were put in contact with 0.03 g of MNPs. Each one of these solutions, together with the concentration of MNPs, was placed in beakers and left in agitation for 30 min at pH = 2 until reaching equilibrium; experiments were carried out in triplicate in order to quantify the error. The results obtained from the readings of the five concentrations of Cr(VI) placed in contact with the concentration of MNPs, in triplicate, were used to perform the linearization of the Langmuir and Freundlich isotherm models.

The Langmuir isotherm was modeled according to the following equation:

(8) $$q_{e}=q_{max}\frac{C_{e}*b}{\left(1+b*C_{e}\right)},$$

where q_e_ represents the amount of adsorbate on the surface at equilibrium (mg/g); C_e_ represents the equilibrium concentration of adsorbate (Cr(VI)) in solution (mg/L); b represents the constant, referring to the adsorption affinity between the nanoparticle and Cr(VI); and q_max_ represents the maximum amount of Cr(VI) per unit mass of nanoparticle to complete the monolayer (mg/g). The numerical values of q_max_ and b are obtained from the slope and intercept, respectively, of the linear form of the isotherm.

The Freundlich isotherm was modeled with the following equation:

(9) $$\log\left(q_{e}\right)=\log\left(K_{f}\right)+\frac{1}{n}\log\left(C_{e}\right)$$

where q_e_ represents the adsorption capacity of magnetite nanoparticles at equilibrium (mg/g); C_e_ represents the Cr(VI) concentration at equilibrium (in mg/L); and K_f_ is the Freundlich counter, which is related to the incorporation of all factors affecting adsorption capacity.

### 3.7. Kinetic Study

The adsorption kinetics of Cr (VI) metal ion can be influenced by the adsorption reaction and mass transfer. In order to identify the controlling mechanism in the adsorption rate of Cr (VI), the experimental data obtained from the equilibrium time were linearized to pseudo-first-order and pseudo-second-order models. The coefficient of determination R^2^ is the parameter, which indicates the best model, and it is obtained from the equation of the line. The highest R^2^ determines the model, which best fits the adsorption process.

## 4. Conclusions

The employment of green synthesis achieved magnetic nanoparticles with a diameter between 73.28 and 162.9 nm. It was also observed that the adsorption process was monolayer, which corresponded to the Langmuir isotherm. The optimum removal results were obtained at pH = 2 and agitation speed = 140 rpm for a contact time = 30 min. Finally, the removal percentage achieved in the tanning effluent was 79.13%, while in the retanning effluent, a value close to 100% removal was reached. Overall, these results demonstrate the efficiency of the proposed method, positioning it as a viable alternative for the industrial treatment of effluents with high concentrations of Cr(VI) from tanneries.

## Figures and Tables

**Table 1:** Characteristics of tanning and retanning effluents.

Stage	Type	Parameter	Value (mg/L)	MPL Value (mg/L)
**Retanning**	In situ	pH	3.567	6.5–9.5
		Dissolved oxygen	5.81	-
		Temperature	18.7	
	Ex situ	Total chromium	1599.14	5
		Hexavalent chromium	4.913	0.5
		Oils and fats	64.9	
		Total suspended solids	53951	1000
**Tanning**	Ex situ	Total chromium	1610.65	5
		Hexavalent chromium	1287.72	0.5
		Oils and fats	15.5	
		Total suspended solids	241	1000
		BODS	199	1000
		COD	4155	

**Table 2:** Precision test results.

Concentration (mg/L)	Absorbance	Cf (mg/L)
0.6	0.18	0.6947705
0.6	0.181	0.7054429
0.6	0.177	0.6627535
0.6	0.179	0.6840982
0.6	0.181	0.7054429
0.6	0.179	0.6840982
Average		0.689
Standard deviation		0.0162
RSD %		0.023

**Table 3:** Results of target readings.

Targets	X	Y
1	0.2	0.131
2	0.4	0.154
3	0.6	0.174
4	0.8	0.192
5	1	0.206
6	1.2	0.228
Average	0.7	0.181
Standard deviation	0.374	0.035
Typical error xy		0.0027
LOD (mg/L)		0.0528
LOQ (mg/L)		0.1760

**Table 4:** Results of the readings for the recovery percentage.

Concentration (mg/L)	Absorbance	Average
0.6	0.174	0.174
	0.174	
	0.174	
0.6	0.173	0.173
	0.173	
	0.173	
0.6	0.171	0.171
	0.171	
	0.172	
Average absorbance		0.173

**Table 5:** Values of the equilibrium parameter (R_L_) derived from the Langmuir model.

R_L_ Value	Type of Isotherm
R_L_ > 1	Unfavorable
R_L_ = 1	Linear
0 < R_L_ < 1	Favorable
R_L_ = 0	Irreversible

**Table 6:** Results of R_L_ to determine the type of isotherm.

C_0_ mg/L	b	R_L_	Average R_L_
0	0.709	1	0.2468
10		0.124	
20		0.066	
50		0.027	
80		0.017	

**Table 7:** Parameters of the isothermal models.

Langmuir	q_max_ (mg/g)	b	R^2^
	1.226	0.709	0.9194
Freundlich	$K_{f}\;(mg1-n\cdot Ln/g)$	n	R^2^
	7.0153	0.1698	0.5621

**Table 8:** Absorbance vs. time results.

Time (min)	Absorbance (nm)			Concentration (mg/L)		
	R1	R2	R3	R1	R2	R3
0	1.722	1.722	1.504	17.1515	17.1515	14.8250
5	0.596	0.956	0.956	5.1345	8.9765	8.9765
10	0.360	0.566	0.360	2.6158	4.8143	2.6158
20	0.054	0.262	0.262	0.0000	1.5699	1.5699
30	0.061	0.083	0.061	0.0000	0.0000	0.0000
60	0.041	0.043	0.043	0.0000	0.0000	0.0000
90	0.034	0.034	0.036	0.0000	0.0000	0.0000
120	0.033	0.035	0.032	0.0000	0.0000	0.0000
150	0.036	0.034	0.032	0.0000	0.0000	0.0000
180	0.033	0.034	0.031	0.0000	0.0000	0.0000
210	0.031	0.032	0.032	0.0000	0.0000	0.0000

**Table 9:** Adsorption capacity (QE) (mg/g) depending on residence time.

Time (min)	Cr(VI) Concentration (mg/L)				Adsorption Capacity (QE) (mg/g)
	R1	R2	R3	Average	
0	17.1515	17.1515	14.8250	16.3760	0
5	5.1345	8.9765	8.9765	7.6958	0.2604
10	2.6158	4.8143	2.6158	3.3486	0.3908
20	−0.6499	1.5699	1.5699	0.8300	0.4664
30	0.0000	0.0000	0.0000	0.0000	0.4913
60	0.0000	0.0000	0.0000	0.0000	0.4913
90	0.0000	0.0000	0.0000	0.0000	0.4913
120	0.0000	0.0000	0.0000	0.0000	0.4913
150	0.0000	0.0000	0.0000	0.0000	0.4913
180	0.0000	0.0000	0.0000	0.0000	0.4913
210	0.0000	0.0000	0.0000	0.0000	0.4913

**Table 10:** Parameters of the kinetic model.

Kinetic Model					
Pseudo First Order			Pseudo Second Order		
q_e_, mg/g	K_1_, m^−1^	R^2^	q_e_, mg/g	K_2_, mg^−1^min^−1^	R^2^
0.2536	0.2227	0.9792	0.6652	0.1962	0.9907

**Table 11:** Chromium concentration.

Chromium Concentration (mL)	Cr(VI) Stock Solution (mL)	H_2_O (mL)
0.0	0.000	10.00
0.2	0.023	9.977
0.4	0.046	9.954
0.6	0.070	9.930
0.8	0.093	9.907
1.0	0.116	9.884
1.2	0.139	9.861

## Data Availability

The findings of this research are supported by data from the corresponding author, M.B., which are available upon reasonable request.

## References

[1] Bharagava R., Saxena G., Mulla S., Patel D. (2018). Characterization and identification of recalcitrant organic pollutants (rops) in tannery wastewater and its phytotoxicity evaluation for environmental safety. Arch. Environ. Contam. Toxicol..

[2] Esparza E., Gamboa N. (2001). Contaminación Debida a la Industria Curtiumbre. Rev. Química.

[3] Sharma P., Singh S., Parakh S., Tong Y. (2022). Health hazards of hexavalent chromium (Cr(VI)) and its microbial reduction. Bioengineered.

[4] Ge F., Li M.-M., Ye H., Zhao B.-X. (2012). Effective removal of heavy metal ions Cd^2+^, Zn^2+^, Pb^2+^, Cu^2+^ from aqueous solution by polymer-modified magnetic nanoparticles. J. Hazard. Mater..

[5] Wang B., Li Q., Lv Y., Fu H., Liu D., Feng Y., Xie H., Qu H. (2021). Insights into the mechanism of peroxydisulfate activated by magnetic spinel CuFe2O4/SBC as a heterogeneous catalyst for bisphenol S degradation. Chem. Eng. J..

[6] Saif S., Tahir A., Chen Y. (2016). Green Synthesis of Iron Nanoparticles and Their Environmental Applications and Implications. Nanomaterials.

[7] Alsaiari N., Alzahrani F., Amari A., Osman H., Harharah H., Elboughdiri N., Tahoon M. (2023). Plant and Microbial Approaches as Green Methods for the Synthesis of Nanomaterials: Synthesis, Applications, and Future Perspectives. Molecules.

[8] Gautam P., Shivalkar S., Banerjee S. (2020). Synthesis of *M. oleifera* leaf extract capped magnetic nanoparticles for effective lead [Pb (II)] removal from solution: Kinetics, isotherm and reusability study. J. Mol. Liq..

[9] Aragaw T., Bogale F., Aragaw B. (2021). Iron-based nanoparticles in wastewater treatment: A review on synthesis methods, applications, and removal mechanisms. J. Saudi Chem. Soc..

[10] Yew Y., Shameli K., Miyake M., Khairudin N., Mohamad S., Naiki T., Lee K. (2020). Green biosynthesis of superparamagnetic magnetite Fe_3_O_4_ nanoparticles and biomedical applications in targeted anticancer drug delivery system: A review. Arab. J. Chem..

[11] Niculescu A.-G., Chircov C., Grumezescu A. (2022). Magnetite nanoparticles: Synthesis methods—A comparative review. Methods.

[12] Aftabtalab A., Chakra C., Sadabadi H., Rao V. (2014). Magnetite nanoparticles (Fe_3_O_4_) synthesis for removal of Chromium (VI) from waste water. Int. J. Sci. Eng. Res..

[13] Hao R., Li D., Zhang J., Jiao T. (2021). Green Synthesis of Iron Nanoparticles Using Green Tea and Its Removal of Hexavalent Chromium. Nanomaterials.

[14] Pacheco-Portugal J.-D. (2019). Evaluación Del Proceso De Biosorción De Cr(VI) Usando Residuos Agroindustriales De La Región Arequipa (Cascarilla De Arroz Y Chala De Maiz). Bachelor’s Thesis.

[15] Cañazaca C., Ccama W. (2017). Biosíntesis De Nanopartículas De Hierro Cero Valente (Nzvi) Usando Hojas De Eucalipto (*Eucalyptus* Sp.) Para La Remoción De Cromo Hexavalente. Bachelor’s Thesis.

[16] Shafey A.E. (2020). Green synthesis of metal and metal oxide nanoparticles from plant leaf extracts and their applications: A review. Green Process. Synth..

[17] Es’haghi Z., Vafaeinezhad F., Hooshmand S. (2016). Green synthesis of magnetic iron nanoparticles coated by olive oil and verifying its efficiency in extraction of nickel from environmental samples via UV–vis spectrophotometry. Process Saf. Environ. Prot..

[18] Fazlzadeh M., Rahmani K., Zarei A., Abdoallahzadeh H., Nasiri F., Khosravi R. (2017). A novel green synthesis of zero valent iron nanoparticles (NZVI) using three plant extracts and their efficient application for removal of Cr(VI) from aqueous solutions. Adv. Powder Technol..

[19] Assi N., Aberoomand-Azar P., Saber-Tehrani M., Husain S., Darwish M., Pourmand S. (2018). Selective solid-phase extraction using 1,5-diphenylcarbazide-modified magnetic nanoparticles for speciation of Cr(VI) and Cr(III) in aqueous solutions. Int. J. Environ. Sci. Technol..

[20] Galán P., Ministerio de Desarrollo Social y Medio Ambiente (1999). Manual para Inspectores Control de Efluentes Industriales.

[21] (2023). International Standard Methods for the Examination of Water and Wastewater.

